# Resilience to maintain quality of intrapartum care in war torn Yemen: a retrospective pre-post study evaluating effects of changing birth volumes in a congested frontline hospital

**DOI:** 10.1186/s12884-020-03507-5

**Published:** 2021-01-07

**Authors:** Josephine Obel, Antonio Isidro Carrion Martin, Abdul Wasay Mullahzada, Ronald Kremer, Nanna Maaløe

**Affiliations:** 1 Médecins Sans Frontières, Saana, Yemen; 2 Department of Obstetrics and Gynaecology, Hvidovre University Hospital, Hvidovre, Denmark; 3 Médecins Sans Frontières, London, UK; 4 Médecins Sans Frontières, Amsterdam, The Netherlands; 5 Global Health Section, Department of Public Health, University of Copenhagen, Copenhagen, Denmark

**Keywords:** Childbirth, Humanitarian response, Quality of care, Yemen, Caesarean section, Adaptive response, Fragile and conflict-affected states, Armed conflict

## Abstract

**Background:**

Fragile and conflict-affected states contribute with more than 60% of the global burden of maternal mortality. There is an alarming need for research exploring maternal health service access and quality and adaptive responses during armed conflict. Taiz Houbane Maternal and Child Health Hospital in Yemen was established during the war as such adaptive response. However, as number of births vastly exceeded the facility’s pre-dimensioned capacity, a policy was implemented to restrict admissions. We here assess the restriction’s effects on the quality of intrapartum care and birth outcomes.

**Methods:**

A retrospective before and after study was conducted of all women giving birth in a high-volume month pre-restriction (August 2017; *n* = 1034) and a low-volume month post-restriction (November 2017; *n* = 436). Birth outcomes were assessed for all births (mode of birth, stillbirths, intra-facility neonatal deaths, and Apgar score < 7). Quality of intrapartum care was assessed by a criterion-based audit of all caesarean sections (*n* = 108 and *n* = 82) and of 250 randomly selected vaginal births in each month.

**Results:**

Background characteristics of women were comparable between the months. Rates of labour inductions and caesarean sections increased significantly in the low-volume month (14% vs. 22% (relative risk (RR) 0.62, 95% confidence interval (CI) 0.45-0.87) and 11% vs. 19% (RR 0.55, 95% CI 0.42-0.71)). No other care or birth outcome indicators were significantly different. Structural and human resources remained constant throughout, despite differences in patient volume.

**Conclusions:**

Assumptions regarding quality of care in periods of high demand may be misguiding - resilience to maintain quality of care was strong. We recommend health actors to closely monitor changes in quality of care when implementing resource changes; to enable safe care during birth for as many women as possible.

## Background

Around 1.2 billion of the world’s population live in fragile and conflict-affected states (FCAS). These states contribute with more than 60% of the global burden of maternal mortality. The Sustainable Development Goals call for specific attention to address maternal health in the realities of armed conflict [1, 2].

In war-affected Yemen, a country that has historically received scarce attention in the health literature, the challenges in providing maternity care are evident. In 2014, Yemen remained with maternal death estimates between 148 and 270 per 100,000 live births, and neonatal deaths between 22 and 26 per 1000 live births [3–5]. Since 2015, Yemen has been in a state of armed conflict with subsequent breakdown of the health services. In 2016, 17% of the public health facilities were out of function and 38% were only partly functional, due to damage of facilities or lack of supplies or human resources [6]. The United Nations have repeatedly issued warnings as demand for humanitarian assistance exceeds available capacity [7, 8].

While there is an urgent need for data to inform the emergency response and prepare for resurrection of the health system, health information systems in Yemen have been unreliable since the beginning of the conflict [9]. Moreover, a systematic review found no studies from Asia and the Middle-East reporting exclusively on maternal health during acute conflict [10]. There is an alarming need for research exploring maternal health service access, quality and adaptive responses during acute crises [11].

Taiz Houbane Maternal and Child Health (MCH) Hospital in Yemen is an example of an adaptive response to mitigate the consequences of armed conflict. The hospital was established by Médecins Sans Frontières (MSF) in November 2015 and pre-dimensioned to serve 650 women in childbirth per month. The dimensioning was based on the estimated need. Staffing, equipment and physical capacity were planned accordingly and in line with MSF’s experience of running frontline hospital services. Since 2015, the hospital, however, experienced a steady increase in demand for services. In August 2017, 1014 births took place, and the facility had provided care 50% above the pre-dimensioned capacity for several months. To mitigate the growing demand for care, a policy was introduced to restrict admissions; it was decided that no new hospital admittances were allowed when bed occupancy reached 100%. Patient seeking care at the facility was at times of full occupancy asked to seek services elsewhere. In cases of severe emergencies, MSF organised transport to alternative MSF facilities in the country or accepted admittance despite full occupancy. Following, as here presented, a research evaluation assessed the restriction’s effects on the quality of intrapartum care and birth outcomes, to provide information for future provision of care and adaptive responses.

## Methods

A retrospective criterion-based audit was conducted from case files of labouring women at Taiz Houbane MCH Hospital in the last month with extreme number of births (August 2017, 1014 labouring women) and the first month where the restriction policy was fully implemented (November 2017, 428 labouring women). Hence, before and after a doubling in provider-to-labouring women ratio.

### Setting

The Taiz Houbane MCH Hospital is located in the Taiz suburb of Houbane in Yemen. Since the outbreak of the war, Taiz city has harboured one of the most active Yemeni frontlines. The hospital serves citizens inhabiting or displaced to the Taiz Governorate (pre-war population of 3.1 million). It is the only facility in the governorate providing comprehensive emergency obstetric and neonatal care (CEmONC) services free of charge, including basic obstetric care, blood transfusions, caesarean sections and other surgical management [12]. The facility also provides antenatal care (ANC), family planning services, paediatric care and malnutrition treatment for children under five years of age. The maternity department is divided into a unit for intrapartum care and clinics for ANC and family planning. The intrapartum care unit is split in two wings and is staffed at daytime with a total of two gynaecological specialist doctors, two non-specialized medical doctors, three nurses and eight midwives. During weekends and nights (17 − 08 hours), the staff composition is identical to daytime, except for only one specialist doctor on duty. All staff are nationals recruited from Yemen, and supervised by an expatriate specialist in obstetrics and gynaecology. The total bed capacity is 45, with 12 low-risk pre-labour/labour beds, six high-risk pre-labour beds for women with severe complications and 27 post-labour beds. Midwives attend to the labouring women, nurses primarily care for the post-partum women, and doctors have responsibilities for both. Hence, if one woman in each labour bed, which corresponds to approximately 650 women in childbirth per month, the birth-attendant-to-labouring-women ratio is 1:1.5 in daytime and 1:1.6 at nights and in weekends (birth attendants include both doctors and midwives).

### Criterion-based audit

The Donabedian framework for assessing quality of care by evaluating structure, process and outcome was applied to ensure a broader understanding of quality than simple birth outcomes [13].

The audit criteria were selected from previous Tanzanian studies, where they were successfully applied [14, 15]. They were modified to suit the MSF guidelines for maternal and newborn care and resemble locally agreed essential practices [16]. Only indicators that were recorded and retrievable from case files were included. Structural resources were assessed by staff numbers, number of labouring women and available supplies, as well as by available background characteristics of the labouring women. Key criteria for the process of intrapartum care delivery included partograph use, intrapartum monitoring of maternal blood pressure, foetal heart rate and labour progress, use of oxytocin augmentation and indications for caesarean section. Birth outcome criteria included rates of stillbirths, intrafacility neonatal deaths and Apgar scores < 7.

All case files were retrieved from the hospital storage where they were filed by month and mode of birth. Case files of births before gestational age of 28 weeks were excluded. Birth outcomes and mode of birth were assessed on all case files in the two months studied. Case files of 250 vaginal births were selected from each month by systematic random sampling to assess the process of care delivery. Case files were piled, the total number was divided by 250 to get the sampling interval. According to this number, every third to fifth file was selected. The file number in the pile from which the count started was determined by drawing of a lot. This process was necessary due to considerable limitations in time and resources, as the data collectors were also busy with clinical work. In addition, all women giving birth by caesarean section were included from both study months to assess the indications for the surgeries.

In accordance with the pre-selected audit criteria, the first author and a Yemeni research assistant extracted data from the case files, which were double-entered into a data collection form in Excel. Any practice not recorded was assumed not being done [17].

For data analysis, the statistical software R was used. Frequencies and percentages were calculated for background characteristics of the labouring women, process of care delivery and birth outcomes. Comparisons between the two periods (high- and low-volume month) were done using Chi-square tests (Fisher’s exact test was used when any category cell count was less than five). Comparisons between two time periods are presented in Tables 1, 2 and 3. Indicators for women in both periods are only presented in the text of the result section. Additionally, we calculated relative risk (RR) with 95% confidence interval (CI) for process and outcome data, considering the exposure to be admission in the high-volume month (August 2017).

**Table 1:** Background characteristics. Comparison of al included women for the in-depth analyses from the high- and low-volume months (250 vaginal births from each month and all caesarean sections). When comparing background characteristics between women with vaginal births and caesarean sections in each of the study months, no significant differences were found, and they are here presented together

	High-volume month *August 2017*	Low-volume month November 2017	*p*-value^a^
	*n (%)*	*n (%)*	
*Of all women*	*(n = 358)*	*(n = 332)*	
**Age**			
< 20 years old	25 (7.1)	29 (8.8)	I 0.801
20–29 years old	153 (43.2)	146 (44.2)	I
30–39 years old	158 (44.6)	138 (41.8)	I
≥ 40 years old	18 (5.1)	17 (5.2)	I
Information missing	4 (-)	2 (-)	
**Parity**			
0	140 (39.1)	106 (31.9)	I 0.064
1–4	161 (44.9)	178 (53.6)	I
≥ 5	57 (15.9)	48 (14.5)	I
Information missing	0 (-)	0 (-)	
**Singleton/multiple gestations**			
Singletons	351 (98.0)	324 (97.6)	I 0.683
Multiple gestations	7 (2.0)	8 (2.4)	I
Information missing	0 (-)	0 (-)	
**Gestational age**			
Extremely preterm (week 28–33)	19 (5.5)	18 (5.5)	I 0.182
Preterm (week 34–36)	27 (7.8)	42 (12.7)	I
Term (37–41 weeks)	291 (84.3)	265 (80.3)	I
Post term (≥ 42 weeks)	8 (2.3)	5 (1.5)	I
Information missing	13 (-)	2 (-)	
**Antenatal care**			
≥ 1 visit	137 (43.6)	152 (52.2)	I 0.034^*ψ*^
Not attended	177 (56.4)	139 (47.8)	I
Information missing	44 (-)	41 (-)	
**Obstetric risk factors in current pregnancy**			
Pre-eclampsia	34 (9.5)	30 (9.1)	I 0.478
Eclampsia	2 (0.6)	4 (1.2)	I
Premature rupture of membrane	38 (10.6)	52 (15.8)	I
Ante-partum haemorrhage	12 (3.4)	11 (3.3)	I
Fever	1 (0.3)	2 (0.6)	I
Other^b^	27 (7.5)	23 (7.0)	I
None	244 (68.2)	208 (63.0)	I
Information missing	0 (-)	2 (-)	
*Of women with parity ≥ 1*	*(n = 218)*	*(n = 226)*	
**Obstetric history**			
Previous death of child/children^c^	53 (26.5)	54 (24.5)	0.646
1 previous caesarean section	30 (13.8)	35 (15.5)	I 0.409
≥ 2 previous caesarean sections	22 (10.1)	15 (6.6)	I
*Of all women*	*(n = 358)*	*(n = 332)*	
**Progress on admission**			
Before active phase of labour^d^	60 (20.0)	55 (19.3)	I 0.683
First stage of active phase of labour	197 (65.7)	180 (63.6)	I
Second stage of labour	43 (14.3)	48 (17.0)	I
Information missing	58 (-)	49 (-)	
**Referrals after failed trial of labour at home**			
Referrals^e^	24 (6.7)	25 (7.6)	I 0.665

*ψ p-value < 0.05*

^a^Fisher exact test

^b^ Other obstetric risk factors: Polyhydramnios (23); Oligohydramnios (14); Suspected chorionamnitis (3); Foetal malformation (1); Bicorn uterus (1); Diabetes (3); Prolonged labour and haematuria prior to admission (1); Hypothyroidism (2); Anaemia (1); First twin delivered prior to admission second still intrauterine (1)

^c^Documentation was insufficient to differentiate perinatal deaths from deaths later in life

^d^Cervical dilation < 4 cm

^e^30 of all 34 women with failed trial of labour at home (88.2%) had received unregulated oxytocin or misoprostol (15/49 missing information regarding prior augmentation)

**Table 2:** Quality of intrapartum care compared between the high- and low-volume months, among 250 randomly selected vaginal births and all caesarean sections in each study month

	High-volume month *August 2017*	Low volume-month *November 2017*	*RR (95% CI)*
	*n (%)*	*n (%)*	
**Labour induction**			
*Of all women, both vaginal births and caesarean sections*	*(n = 358)*	*(n = 332)*	
Labours induced^a^	*50 (14.0)*	*74 (22.4)*	*0.62 (0.45–0.87)*^*ψ*^
**Instrumental deliveries**			
*Of all women delivering in the two months*	*(n = 1014)*	*(n = 428)*	
Caesarean sections^b^	*108 (10.7)*	*82 (19.2)*	*0.55 (0.42–0.71)*^*ψ*^
*Of all included women with vaginal deliveries*	*(n = 250)*	*(n = 250)*	
Instrumental vaginal deliveries	*2 (0.8)*	*4 (1.6)*	*0.50 (0.09–2.73)*
**Overall partograph use**			
*Of women in first stage active phase of labour and vaginal delivery*	*(n = 190)*	*(n = 190)*	
No correct plot on the partograph’s alert line	38 (20.0)	29 (15.3)	1.31 (0.84–2.03)
**Foetal surveillance**			
*Of women with vaginal delivery and positive foetal heart rate on admission*	*(n = 187)*	*(n = 186)*	
> 1 hour between fetal heart rate readings during active labour	50 (26.7)	42 (22.6)	1.18 (0.83–1.69)
**Labour progress**			
*Of women with vaginal delivery where first stage of active labour exceeded 4 hours*	*(n = 68)*	*(n = 51)*	
> 4 hours between two cervix recordings	10 (14.7)	3 (5.9)	2.50 (0.72–8.62)
*Of women in first stage active phase of labour and vaginal delivery*	*(n = 190)*	*(n = 190)*	
Action line crossed	5 (2.7)	1 (0.5)	5.0 (0.59–42.40)
*Of all women with vaginal delivery excluding inductions*	*(n = 220)*	*(n = 193)*	
Oxytocin augmentation, total use^c^	51 (23.1)	51 (26.4)	0.88 (0.63–1.23)
**Maternal vital signs** *Of all women with vaginal delivery*	*(n = 250)*	*(n = 250)*	
None or > 4 hours between blood pressure readings	75 (30.0)	70 (28.0)	1.07 (0.81–1.41)
**Indications for caesarean sections** *Of all women with delivery by caesarean section*	*(n = 108)*	*(n = 82)*	
Prolonged labour^d^	30 (28.3)	23 (28.0)	0.99 (0.62–1.57)
Foetal distress^e^	12 (11.3)	13 (15.9)	0.70 (0.33–1.45)
Two or more previous caesarean sections	22 (20.8)	14 (17.1)	1.19 (0.65–2.19)
Malpresentation	15 (14.2)	5 (6.1)	2.28 (0.86–6.01)
One previous caesarean section and risk of rupture	10 (9.4)	3 (3.7)	2.53 (0.72–8.90)
Others^f^	19 (17.5)	24 (29.3)	0.60 (0.35–1.02)

*ψ p-value < 0.05*

^a^First choice induction method: In August 2017, 7/50 (14%) were induced by artificial rupture of membranes, 24/50 (48%) by misoprostol and 19/50 (38%) by oxytocin. In November 2017, 17/74 (23%) were induced by artificial rupture of membranes, 35/74 (47%) by misoprostol and 22/74 (30%) by oxytocin. The most common indications for induction were *pre-eclampsia, pre-labour rupture of membranes and postterm, and there were no significant differences in the frequencies of indications in the months studied (p = 0.63).*

^b^In 6/108 (6%) and 4/82 (5%), respectively, caesarean section was performed after diagnosed intrauterine foetal death.

^c^In 26/220 (12%) and 19/193 (10%), respectively, oxytocin augmentation was initiated before crossing the action line.

^d^In 19/30 (63%) and 19/23 (83%), respectively, the action line was either not yet crossed or the partograph unused when deciding on caesarean section due to prolonged labour, and in 16/30 (53%) and 7/23 (30%) oxytocin augmentation had not been tried.

^e^In 6/12 (50%) and 6/13 (46%), respectively, last FHR was recorded in the normal range (110–160 bpm).

^f^Other indications for caesarean sections placenta previa, severe antepartum haemorrhage, cord prolapse, rupture of uterus, reduced foetal movement, unclear indications

**Table 3:** Perinatal outcomes in the high- and low-volume months

	High-volume month *August 2017*	Low-volume month *November 2017*	RR (95% CI)
***Perinatal deaths Of all births***	*(n=1034)*	*(n=436)*	
	*n (per 1000 total births)*	*n (per 1000 total births)*	
Stillbirths, in total	*34 (32.9)*	*19 (43.6)*	0.76 (0.44-1.32)
Missing information	*0(-)*	*0 (-)*	
Intra-hospital stillbirths^b^	*10 (9.7)*	*1 (2.3)*	4.22 (0.54-32.84)
*Missing information*	*0 (-)*	*2 (-)*	
***Perinatal deaths*** ***Of all live births***	*(n=1000)*	*(n=417)*	
	*n (per 1000 live births)*	*n (per 1000 live births)*	
Intra-hospital neonatal deaths	*6 (6.0)*	*7 (16.8)*	0.36 (0.12-1.06)
Missing information	*0 (-)*	*0 (-)*	
***Perinatal outcomes of all births in sample selected for in depth quality audit***	*(n=365)*	*(n=340)*	
	*n (%)*	*n (%)*	
**Birthweights**			
<1500 gram	11 (3.1)	9 (2.7)	1.13 (0.47-2.69)
1500-2499 gram	70 (19.4)	62 (18.3)	1.04 (0.77 – 1.42)
≥ 2500 gram	279 (77.5)	267 (79.0)	0.99 (0.91 – 1.07)
Information missing	5 (-)	2(-)	
**Congenital malformations**^a^			
Congenital malformation observed during admission	3 (0.8)	3 (0.9)	0.92 (0.19-4.51)
No malformations observed	362 (99.2)	333 (99.1)	1.01 (0.91-1.12)
Information missing	0 (-)	4 (-)	
	*n (per 1000 total births)*	*n (per 1000 total births)*	
**Perinatal deaths during admission**			
Stillbirths, in total	16 (43.8)	13 (38.2)	1.15 (0. 56-2.35)
Information missing	0 (-)	0 (-)	
Intra-hospital stillbirths^b^	5 (13.7)	1 (3.0)	4.63 (0.54-39.43)
Information missing	0 (-)	2 (-)	
	(*n*=349) *n (per 1000 live births)*	*(n=327)* *n (per 1000 live births)*	
Apgar score 1-6 at 5 minutes	18 (51.6)	18(55.2)	0.93 (0.49-1.76)
Information missing	0 (-)	1 (-)	
Intra-hospital neonatal deaths	5 (14.3)	7 (21.4)	0.67(0.21-2.09)
Information missing	0 (-)	0 (-)	

^a^In total, 5 of the congenital malformations were not further described. One was a cleft palate

^b^Stillborn babies with positive foetal heart rate upon admission

### Ethical considerations and funding

Data were collected retrospectively and recorded and stored in an anonymized form without patient or provider identifiable information. This research fulfilled the exemption criteria set by the Médecins Sans Frontièrs’ Ethics Review Board (ERB) for a posteriori analyses of routinely collected clinical data and thus did not require an elaborate ERB review. There was no functioning ethics review board in Yemen, and the study was conducted with permission from the Medical Director of Médecins Sans Frontièrs’ (MSF) Operational Centre in Amsterdam (OCA), who oversees all field operations of MSF – OCA including the Taiz Houbane MCH Hospital in which this data was collected. MSF supported data collection, analysis and publication, with no relation to industry or multi- or bilateral agencies.

## Results

The total number of women giving birth at Taiz Houbane MCH Hospital was 1014 in August 2017 and 428 in November 2017, of which 11% (108/1014) and 19% (82/428) were delivered by caesarean section, respectively. By randomly selecting 250 women with vaginal births from each month studied to assess quality of care further, 25% of women in August were included for the in-depth analysis and 58% in November. MSF did not collect data on number and characteristics of women turned away after the restriction policy was implemented.

Background characteristics of labouring women were compared between the 250 women with vaginal birth in each period selected for in-depth analysis as well as for all the caesarean sections. There were no significant differences between the subgroups of women with vaginal births and caesarean sections, and they are here presented as one group for each study month (Table 1). Proportion of women attending a minimum of one ANC visit was lower in the low-volume month compared with the high-volume month (44% (137/358) versus 52% (152/332), *p* = 0.034). No other background characteristics showed significant difference. In brief, of all included women that gave birth in Taiz Houbane MCH Hospital in both study months, a total rate of 34% (236/690) had pre-existing obstetrical risk factors in the current pregnancy. Among all, 36% (246/690) were nulliparous, 15% (105/690) were para five or more, and 8% (54/690) were below the age of 20 years. The total number of babies born extremely premature (under 28 weeks of gestation) was not recorded, but the proportion of all babies born with a birthweight under 2.5 kg in the low volume month was 18.0%, compared with 17.3% in the high volume month. Among multiparous women, 24% (107/444) had previously suffered from the death of a child. Of all women, 7% (49/690) were admitted after failed trial of labour at home. Among these, where information on pre-hospital augmentation was available, 88% (30/34) had received unregulated oxytocin or misoprostol at home.

### Structure

The number of midwives, nurses and doctors were constant throughout the two time periods, and there were no major changes in equipment, physical structures or drugs available throughout the two months audited. At all times, the hospital was able to provide all CEmONC signal functions. The ratio of birth-attendant-to-labouring-women in the high-volume month of August and the low-volume month of November was 1:2.7 and 1:1.2 in daytime. The number of staff outside weekday daytime hours was slightly lower with a ratio of birth-attendant-to-labouring-women of 1:3.0 in August and 1:1.3 in November.

### Process of care delivery

Among all women giving birth at Taiz Houbane MCH Hospital in the study months (*n* = 1014 in August and n = 428 in November), the rate of caesarean sections differed significantly with 10.7% (108/1014) and 19.2% (82/428), respectively (RR 0.55, 95% CI 0.42–0.71). No differences in distribution of indications for the caesarean sections were found between the two study months, nor any difference in the rate of instrumental births. Among all included women for in-depth audit, significantly more women were induced in the low-volume month (50/358 (14%) and 74/332 (22%); RR 0.62, 95% CI 0.45–0.87). Among the included women giving birth vaginally (n = 250 in each study month), there were no differences between the two months in achieving the additional audit criteria related to overall partograph use, foetal surveillance during labour, detection and reaction to slow progress of labour and monitoring maternal vital signs (Table 2). Among caesarean sections due to prolonged labour in the high- and low-volume month, in 63% (19/30) and 83% (19/23) the action line was either not crossed or the partograph unused when deciding on the surgery, and in 53% (16/30) and 30% (7/23) oxytocin augmentation had not been tried. Among caesarean sections due to foetal distress, half (6/12 and 6/13) were performed with the foetal heart rate documented in the normal range (110–160 beats per minute).

Among women admitted before or during first stage of active labour, 20% (38/190) and 15% (29/190) did not have a correct plot of labour progress on the partograph’s alert line (RR 1.31, 95% CI 0.84–2.03), and if first stage of labour exceeded four hours, 15% (10/68) and 6% (48/51) did not have the cervical dilatation re-plotted (RR 2.50, 95% CI 0.72–8.62). When excluding labour inductions, oxytocin augmentation was applied in 23% (51/220) and 26% (51/193) (RR 0.88, 95% CI 0.63–1.23), of which oxytocin was initiated prior to crossing the partograph’s action line in 12% (26/220) and 10% (19/193) (RR 1.22, 95% CI 0.70–2.13) (Table 2).

### Birth outcomes

There were not significant differences between the two study periods with regard to the birth outcomes assessed (Table 3). Among all women giving birth in the study months (i.e. 1014 in August 2017 and 428 in November 2017), the stillbirth rate was 32.9 and 43.6 per 1000 total births, respectively (RR 0.76, 95% CI 0.44–1.32), of which 9.7 and 2.3 per 1000 total births occurred after admission to the hospital (RR 4.22 95% CI 0.54–32.84). The intrafacility neonatal death rate in the high- and low-volume month was 6.0 and 16.8 per 1000 live births (RR 0.36 95% CI 0.12–1.06). There were no maternal deaths in the study population (Table 3).

Among the offspring of the included women in the in-depth analyses (i.e. 250 vaginal births in each study month and all caesarean sections), there were comparable occurrence of low Apgar score between the months.

## Discussion

In Taiz Houbane MCH Hospital in a Yemeni warzone, a restriction in admissions led to halving in the number of women giving birth, from > 1000 to < 500 births per month. Surprisingly, the study was unable to show improvements in quality of care or birth outcomes after the restriction. However, rates of labour inductions and caesarean sections increased significantly in the low-volume month.

### Strengths and limitations

Criterion-based audits are a pragmatic and low-cost approach to address quality of intrapartum care and birth outcomes in low-resource crisis settings, where attention to providing care is prioritized above more time- and resource-consuming study designs. Yet, while there were simply no more time available for data collection, the study’s sample size limits its ability to detect smaller differences in birth outcomes, and the absent differences found between the two periods studied may be due to lack of power. However, similarities across the periods in achieving the audit criteria for intrapartum care argue against this.

The results are limited to the two months studied and cannot be used to predict the possible effects of high work-pressure on quality of care and birth outcomes for a lengthy time period. While a multiple interrupted time series design with several time points for data collection before and after the restriction policy would have strengthened the design, this was not feasible due to the recourse restrictions of the study. Furthermore, the assumption that any practice not documented was not performed could bias the results towards showing poorer intrapartum care than actually provided. Conversely, there is also a risk of non-performed care being documented as performed.

While the comparable background characteristics among labouring women are reassuring, external confounding factors may have affected the quality of intrapartum care and birth outcomes. Notably, women in the low-volume month had a significantly higher attendance of at least one ANC visit, which might have improved birth outcomes. Moreover, additional characteristics of the background population and socioeconomic data of women seeking care would have allowed a better understanding of the access to care. Unfortunately, no data was available on women failing to access care after introduction of the restriction policy.

As in other FCAS, the humanitarian response in Yemen operates under very difficult conditions, and the decision to scale up or down on intake of beneficiaries served is dependent on multiple factors beyond quality of care, which we were not able to include in this study (e.g. occupational health, supplies, funding and security risks). Also within maternity care provision, other practices that could likely be influenced by changes in birth volume could not be assessed as not clearly recorded in case files, such as management of postpartum haemorrhage, skin-to-skin contact and breastfeeding.

### Interpretation

In November 2017, where the number of births was below pre-dimensioned capacity, the study disclosed a doubling of the caesarean section rate and a simultaneous increase in labour inductions, but no other differences in quality of intrapartum care and birth outcomes. This was surprising. Yet, the ability to provide good quality CEmONC services despite a high and ever-increasing caseload is similar to data from an MSF run hospital in a conflict-affected province of Afghanistan [18]. When conducting our study, the Taiz Houbane MCH Hospital had already worked above the pre-dimensioned capacity during the previous three months, and both studies demonstrate the impressive resilience of health workers to cope with extreme workloads.

Importantly, our study did not assess caring support and working conditions for the staff, which might have deteriorated if the high birth loads had persisted for a longer time [18]. As reported from busy maternity units in sub-Saharan Africa, workload far beyond capacity may lead to demotivation, burnout and impaired performance among providers [19–22]. On the other hand, the working conditions at the MSF run hospitals may, even when congested, be of higher quality than in governmental hospitals within fragile, resource constrained healthcare systems; regarding resources, leadership, accountability, teamwork, guidance and transparency. Furthermore, compared to busy low-resource hospitals in East Africa with workloads similar to the high-volume month, the quality of care provided at these MSF run hospitals is much higher, and the intra-hospital stillbirth rates of 2.3–9.7 much lower [20, 23–27]. This may in itself be a positive driver among staff to keep providing quality care even when working 50% above capacity. Another explanation could be that the birth-attendant-to-labouring-women ratio at these MSF-run hospitals remained above an unknown critical threshold for deterioration in quality of care.

Though the quality of care at the Taiz Houbane MCH Hospital may be encouraging, it was not optimal. Among vaginal births, 18% took place without even one correct plot on the partograph, in a quarter of cases foetal heart monitoring during first stage of labour occurred less frequently than every hour, and 29% of women did not have their blood pressure measured at least every four hours. Moreover, a concerning issue is the increase in labour inductions and caesarean sections in the low-volume month. Among caesarean sections due to prolonged labour, 30% were carried out in women who did not have at least one correct plot on the partograph, and in 43% of these cases augmentation with oxytocin was not tried. Further studies are called for to assess this potential over-medicalization. Notably, interim practice led by humanitarian aid and provided by local health workers may become institutionalized into a new standard practice, also after the conflict. This was for instance seen at a Tanzanian district hospital hosting a refugee camp during the Rwandan genocide. Here, the caesarean section rate doubled during this period, and it never reduced again – but maternal and perinatal outcomes did not improve and surgical complications increased [28].

Literature is scarce on quality of care in CEmONC facilities in FCAS [9, 18]. In contrast to the low intrafacility stillbirth rates, rates of stillbirths with intrauterine foetal death on admission of 32.9 and 43.6 per 1000 total births are unacceptably high, and similar to the Afghani MSF hospital (29 per 1000 total births) [18]. Also, 24% of multiparous women in the present study suffered from previous loss of a child. These findings suggest severe substandard health system performance at the population level and large unmet need for services. Rates of facility births in Yemen are traditionally low, with approximately 30% of women giving birth in health facilities in 2013 [3]. Assuming that women who opt for a facility birth are more likely to attend ANC, the finding that less than half of women in the current study had attended ANC becomes even more worrying, and likely reflects the war-related reduction in service availability. In comparison, a pre-war house-hold survey from Taiz Governorate reported that 66% of women attended ANC at least once [3]. Another ‘symptom’ of the limited health system, which is similarly reported from conflict-affected regions of Pakistan, [29] is the problematic self-administration of intravenous oxytocin among Yemeni women, which can lead to uterine hyperstimulation, foetal death, uterine rupture and bleeding after birth [30].

## Conclusions

In a crisis situation, resource constrained humanitarian actors are faced with the dilemma of providing good care for fewer or less for many, [18] and evidence is warranted to assist in this decision-making [10]. While the decision by humanitarian actors to limit or increase the number of beneficiaries served rely on multiple factors beyond quality of care, quality of care is typically raised as a key concern. This study argues that changes in quality of care may not be as obvious as assumed when facing these dilemmas. We recommend health actors to closely monitor changes in quality of care indicators when implementing changes in resources, and to explore minimal provider-to-labouring-woman ratios and optimal organizational structures to provide safe and respectful care during birth for as many women in need as possible.

## Data Availability

The dataset used and analysed during the current study are available from the corresponding author on reasonable request.

## Abbreviations

ANC: Antenatal care
CEmONC: Comprehensive emergency obstetric and neonatal care
CI: Confidence interval
ERB: Ethical Review Board
FCAS: Fragile and conflict-affected states
MCH: Maternal and Child Health
MSF: Médecins Sans Frontières
RR: Relative risk
